# Fluorescence-Based Binding Characterization of Small Molecule Ligands Targeting CUG RNA Repeats

**DOI:** 10.3390/ijms23063321

**Published:** 2022-03-19

**Authors:** Zhihua Chang, Ya Ying Zheng, Johnsi Mathivanan, Vibhav A. Valsangkar, Jinxi Du, Reham A. I. Abou-Elkhair, Abdalla E. A. Hassan, Jia Sheng

**Affiliations:** 1 Department of Chemistry and The RNA Institute, University at Albany, State University of New York, 1400 Washington Avenue, Albany, NY 12222, USA; zchang@albany.edu (Z.C.); yzheng21@albany.edu (Y.Y.Z.); jmathivanan@albany.edu (J.M.); vibhavvalsangkar@gmail.com (V.A.V.); jdu4@albany.edu (J.D.); 2 Applied Nucleic Acids Research Center & Chemistry Department, Faculty of Science, Zagazig University, Zagazig 44523, Egypt; riham31@yahoo.com

**Keywords:** fluorescence spectroscopy, RNA-ligand binding, microsatellite RNA expansions, CUG repeat, aminoglycosides

## Abstract

Pathogenic CUG and CCUG RNA repeats have been associated with myotonic dystrophy type 1 and 2 (DM1 and DM2), respectively. Identifying small molecules that can bind these RNA repeats is of great significance to develop potential therapeutics to treat these neurodegenerative diseases. Some studies have shown that aminoglycosides and their derivatives could work as potential lead compounds targeting these RNA repeats. In this work, sisomicin, previously known to bind HIV-1 TAR, is investigated as a possible ligand for CUG RNA repeats. We designed a novel fluorescence-labeled RNA sequence of r(CUG)_10_ to mimic cellular RNA repeats and improve the detecting sensitivity. The interaction of sisomicin with CUG RNA repeats is characterized by the change of fluorescent signal, which is initially minimized by covalently incorporating the fluorescein into the RNA bases and later increased upon ligand binding. The results show that sisomicin can bind and stabilize the folded RNA structure. We demonstrate that this new fluorescence-based binding characterization assay is consistent with the classic UV *T_m_* technique, indicating its feasibility for high-throughput screening of ligand-RNA binding interactions and wide applications to measure the thermodynamic parameters in addition to binding constants and kinetics when probing such interactions.

## 1. Introduction

Pathogenic microsatellite RNA expansions are toxic and have been associated with many untreatable neurodegenerative diseases [1], including myotonic dystrophy (DM) [2], amyotrophic lateral sclerosis (ALS) [3], and Huntington’s disease (HD) [4]. These RNA expansions are thought to cause disease through two major mechanisms, which are not mutually exclusive: loss or gain of protein functions due to expansion and translation of RNA repeats (e.g., the disease-causing CAG expansions resulting in the production of proteins containing expanded polyQ tracts), and RNA toxic gain-of-function [5]. It is well known that RNA gain-of-function dominates in a series of diseases including myotonic dystrophy types 1 and 2 (DM1 and DM2), fragile X-associated tremor ataxia syndrome (FXTAS), and spinocerebellar ataxia 8 (SCA8) [6]. In addition, this mechanism also likely underlies SCA types 10 and 12 and Huntington’s disease-like 2 (HDL2) [7]. In a typical disease model, the RNA repeats, which are thought to form hairpin-like structures, often disrupt the functions of specific RNA-binding proteins [8,9]. These proteins have been demonstrated as the regulators of many RNA processes in both nucleus and cytoplasm such as alternative splicing, mRNA stability, translation, and mRNA localization and editing [10,11]. Sequestration of these RNA-binding proteins by the expansion of RNA results in disruption of their normal functions and a series of consequent downstream cellular effects that have been linked to more human diseases.

**Figure 1:**
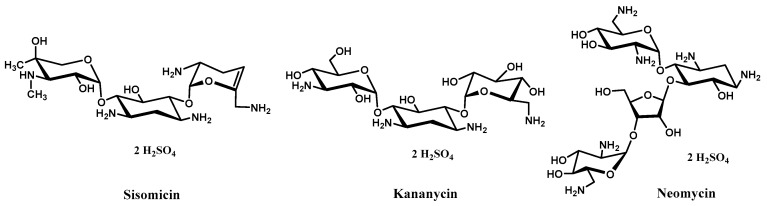
Structures of sisomicin, kanamycin, and neomycin as typical aminoglycosides.

Therapeutics that eliminate or neutralize these RNA repeats hold great promise for the treatment of many neurodegenerative diseases since they are fatal without the treatments available today. Different strategies have been developed targeting these pathogenic RNA repeat sequences including antisense oligonucleotides (ASOs) [12,13,14,15] and small molecule-based binders [16,17,18,19,20,21,22,23,24,25,26,27,28,29,30]. Although great progress has been made in the ASO therapeutic direction, numerous challenges still remain especially on addressing the binding specificity, long-term biostability, delivery to tissues of interest, and the immune system related toxicity, as well as the achievement of the optimal balance between high potency and low off-target effects in different RNA microsatellite targeting [1]. The second strategy involves small molecules that can interfere with the interaction of expanded nucleotide repeats and their connate RNA-binding protein partners. FDA-approved aminoglycoside antibiotics (Figure 1) have been screened for drug repurposing and several studies have shown that some aminoglycosides and their derivatives might be able to work as potential binders of RNA repeats, e.g., neomycin and kanamycin for either DM1 or DM2 [17,19,21,25]. Despite the common issues such as relatively poor binding specificity and low hit rates in screening for lead compounds, the pool of small molecules that target RNA repeats has been largely expanded through rational drug design and screening approaches in recent studies [19,20,21,22,23,24,25,26,27,28,29,30].

**Figure 2:**
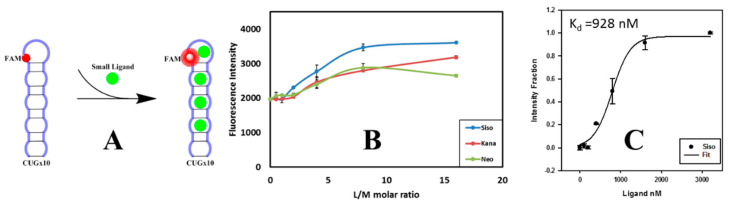
Aminoglycoside comparison experiments: (**A**) ligand binding scheme; (**B**) binding comparison of three aminoglycosides targeting FAM modified r(CUG)_10_ with ligand to RNA molar ratios—L/M at 0, 0.5, 1, 2, 4, 8, and 16; and (**C**) sigmoidal fitting of sisomicin binding data and calculated K_d_ value.

Identification of small ligands targeting these pathogenic RNA repeat sequences is therefore of great significance for the development of potential therapeutics to treat these diseases. In this work, we investigated sisomicin (a binder of HIV-1 TAR [31]) as a possible ligand targeting pathogenic CUG RNA repeats with two reported positive controls, kanamycin and neomycin. By designing a FAM modified 10-repeated CUG sequence r(CUG)_10_ as the RNA template to simulate disease-causing CUG repeat expansions in DM1 (Figure 2A), we applied fluorescence-based measurement to study ligand binding targeting these RNA repeats. Fluorescence techniques are fast, highly sensitive, and can be operated in cost effective and high-throughput fashions, e.g., microplate reader. Unlike most cases of FAM labeling either at 5′ or 3′ end giving it free access in solution, our design minimizes the fluorescence by covalently incorporating fluorescein into the RNA repeat sequence to improve sensitivity and reduce false-positive readings. In comparison to other approaches, such as fluorescence resonance energy transfer (FRET) [32,33,34,35], fluorescent indicator displacement (FID) [36], and multi-step assays [37], the microplate assay developed from this FAM modified RNA template is simple and fast with high sensitivity for reducing false-positive readings. By employing this assay, we found that sisomicin has the highest binding affinity towards CUG RNA repeats among the three tested aminoglycosides.

The second part of our work is focused on characterizing the interaction between sisomicin and the FAM modified CUG RNA template. We were particularly interested in the thermal stability of this interaction and designed a fluorescence thermal melting assay. Conventional UV *T_m_* alone provides very limited conformational information for these studies, thus requiring the incorporation of other techniques to improve the overall mechanistic understanding, e.g., UV optical measurement assisted by a fluorescence-based assay for the study on RNA three-way multibranch junctions [38]. Similarly, a CD study featuring CD thermal melting supplemented with a fluorescence binding characterization and an additional confirmation of UV absorption presented profound details on ligand binding with human telomeric G-quadruplexes [39]. Despite decades of efforts on fluorescent-probing nucleotide thermal denaturation [40,41,42,43,44], challenges such as distorted/curved baselines and inconsistencies with standard UV melting data, still remain. To address these fundamental issues, researchers have developed different strategies and have made promising progress. In a study on DNA bulge conformation [41], 2-aminopurine (extinction coefficient E_λmax_ 3600 M^−1^cm^−1^ and quantum yield-water 0.68) was used to introduce the bulge perturbations showing improved baselines. Another strategy involving the concepts of a molecular beacon (MB) or fluorophore/quencher pair [42,43,44] achieved much success in baseline correction and determination of accurate thermodynamic parameters.

However, these strategies are not desirable for probing the interactions of small molecules targeting RNA repeats due to their high salt conditions and low sensitivity to detect fine RNA conformational changes caused by small molecule binding. The novel fluorescence melting assay that we have developed highlighted the covalent incorporation of 6-FAM (extinction coefficient E_λmax_ 75,000 M^−1^cm^−1^ and quantum yield 0.9) into the CUG repeated sequence for better sensitivity and avoided high salt conditions with more accurate binding characterizations. Our assay adopted an indirect strategy on the accurate *T_m_* acquisition by verifying the existence of two regions for the dissociation of the sisomicin-RNA complex at the actual *T_m_*. The detailed binding characterization was conducted at 20–65 °C, showing that sisomicin stabilized the folded RNA structure upon ligand binding and, furthermore, these results were confirmed by CD spectra. We carefully compared our fluorescence *T_m_* data with traditional UV *T_m_* results and explained the discrepancy between them in detail. By applying the Van ’t Hoff plot to study the dissociation of the RNA-ligand complex at 20–65 °C, we demonstrated that the new approach of *T_m_* measurement is consistent with the traditional UV optical melting technique. This implies that our fluorescence thermal denaturation assay is suitable for broad applications to probe RNA-ligand interactions, comprehensive binding characterization, measurements of the thermodynamic parameters, and determining binding constants and kinetics. This binding characterization assay can also be applied for identifying ligands targeting other microsatellite RNA expansions with high throughput fashion when corresponding modified RNA repeat sequences are employed as templates and targets.

## 2. Results

### 2.1. Aminoglycoside Comparison Study

We constructed a CUG RNA repeats analog by covalently incorporating FAM into the r(CUG)_10_ sequence: 5′-CUGCUGCUGCUGCfamUGCUGCUGCUGCUGCUG-3′. The sequence was designed in a way that the disturbance from FAM has minimum to no effect on the small molecules and RNA repeats interaction. If self-folded, the fluorescein would be located in the hairpin loop as shown in Figure 2A. This FAM modified RNA strand was synthesized to mimic cellular RNA repeats and labeled as FM1 CUG × 10. Using FM1 r(CUG)_10_ as the RNA template, we investigated small molecules targeting CUG RNA repeats, especially some aminoglycosides. We hypothesized that sisomicin, previously reported to bind HIV-1 TAR [31], could also bind CUG RNA repeats, and compared sisomicin binding with two known positive controls—kanamycin and neomycin using fluorescence microplate assay. All three aminoglycosides were tested to confirm no intrinsic fluorescence and the dramatic fluorescence changes come from the ligand-RNA binding complex, not from the FAM affecting fluorescence with ligand interaction. The comparison results shown in Figure 2B indicated that with a higher fluorescence response, sisomicin has a better binding affinity toward CUG RNA repeats compared with kanamycin and neomycin, with a calculated K_d_ value of 928 nM (Figure 2C).

### 2.2. Fluorescence Titration Study at Room Temperature

**Figure 3:**
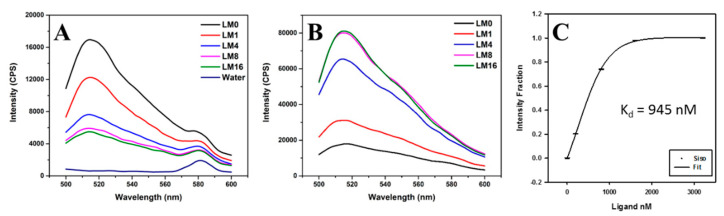
Fluorescence titration using sisomicin as titrant against annealed FM1 CUG × 10 RNA repeats at 25 °C: (**A**) Fluorescence spectra of control experiments with DI water only and 5 nM of 6-FAM water solution titrated by sisomicin at siso-to-FAM molar ratios of 0, 1, 4, 8, and 16; (**B**) fluorescence spectra with ligand to RNA molar ratios—L/M at 0, 1, 4, 8, and 16; (**C**) K_d_ value obtained from sigmoidal fitting; annealed FM1 CUG × 10 RNA sample concentration 200 nM and volume size 150 µL; ligand addition volume size 1.5 µL; fluorescence setting: excitation at 485 nm, emission at 500–600 nm, and slit width of 3 nm.

As our microplate assay is more suitable for drug screening operated in a high-throughput fashion, further mechanistic investigations on ligand binding towards RNA repeats require a more sensitive technique in terms of fluorescence measurement. We then selected sisomicin as the model ligand and performed a detailed binding characterization of CUG RNA repeats on a fluorometer using fluorescence titration, which is similar to our fluorescence thermal melting assay except at room temperature only. The fluorescence titration experiment was carried out with sisomicin targeting annealed FM1 CUG × 10 RNA repeats. Sisomicin additions were prepared with different concentrations (1.5 µL) to achieve equivalent molar ratios as follows: 0, 1, 4, 8, and 16. 6-FAM has an emission maximum of 517 nm, which was not affected by the Raman scattering peak at 580 nm in water solution samples shown in Figure 3A. Interestingly, our control experiments with 5 nM of 6-FAM in water revealed that sisomicin titration decreased the fluorescence intensity of 6-FAM. At room temperature (Figure 3B), the emission spectra of RNA repeats showed no shift with increasing concentration of ligand. This observation, together with the reduction in fluorescence intensity of 6-FAM due to sisomicin additions, further confirms that the increase of fluorescence upon ligand binding is caused by the ligand-RNA interaction, implying that the FAM is not involved in the binding between sisomicin and RNA repeats. Increasing the equivalent molar ratios is similar to a concentration dependent study investigating the binding effects of ligand concentration: the lowest emission curve appeared at L/M = 0; when increasing sisomicin concentration, emission curves at L/M = 8 and 16 became overlapped and were the highest, indicating that the binding was already saturated after L/M = 8. K_d_ was calculated as 945 nM through a sigmoidal fitting. These results are consistent with the aminoglycoside comparison data conducted at room temperature in Figure 2B,C, validating our fluorescence microplate assay as an efficient and reliable method.

### 2.3. UV Thermal Melting Study

Continuing with the sisomicin as the titrating ligand, we performed UV-melting temperature (*T_m_*) studies to characterize the thermal stability of the FM1 CUG RNA template and its interaction with sisomicin. Different CUG repeats (in the supporting information Tables S1 and S2, and Figure S3), i.e., native r(CUG)_7_ as r7, native r(CUG)_10_ as r10, and FM1 r(CUG)_10_ as r10FAM, were synthesized to conduct the thermal stability and structural comparisons. It has been demonstrated that the RNA double helix upon binding with a ligand can usually increase the *T_m_* value, indicating enhanced stability of the RNA structure [45]. UV melting studies were conducted to show the ligand concentration dependency of RNA repeats (Figure S3G,H), revealing a slight improvement in the thermal stability after interacting with sisomicin. In comparison, r7 shows a different *T_m_* behavior from r10 (r10 and r10FAM): a 4-degree jump to 64 °C at L/M = 1, followed by a decrease back to 62 °C as L/M increased, suggesting that the annealed r7 strand might possess a different secondary structure from the r10 ones.

The variety of RNA repeat templates and different RNA concentrations used in thermal denaturation studies enable us to examine the following three factors on *T_m_*: (a) RNA concentration; (b) RNA modification; and (c) number of CUG repeats. For factor (a), roughly tripling r10FAM concentration from 1.5 µM to 5 µM led to a slight increment on *T_m_* by less than 2 °C, and normalized melting curves remained overlapped in most cases, indicating that RNA concentration effect is trivial in the experimental conditions, and it does not affect the thermal denaturation behavior of RNA. For factor (b), covalently coupling fluorescein in between bases C and U at the 5th CUG repeat in r10 introduced a perturbation to the local structure and caused a small shift of the melting curve towards the left and a decrease of 2 °C on *T_m_*. The results in Figure S3 for factor (c) surprisingly contradicted what we expected. Adding three more CUG repeats into the r7 sequence makes the strand become r10, with a total 9 nt longer. If r7 and r10 form similar secondary structures, one would expect that a melting curve shifts to the right with a higher *T_m_* upon transitioning from r7 to r10. However, we observed that instead of a higher *T_m_*, r10 showed a lower *T_m_* in all cases, although r7 was on the left side of r10 in each UV melting plot. This indicates the existence of different secondary structures between the two strands: r7 may form a normal duplex containing 14 GC base pairs; while r10 forms a hairpin with a four-nucleotide hairpin loop and 9 GC base pairs in its stem, which would align with our self-folded hypothesis of the r10 sequence in the scheme of Figure 2A.

### 2.4. Fluorescence Thermal Melting Study

The above UV *T_m_* results inspired us to go for a fluorescence thermal denaturation study on the interaction of sisomicin-RNA repeats using FAM-modified r(CUG)_10_. Before proceeding further, we carefully examined the factors that could affect the fluorescence readings under the elevated temperature conditions for this thermal denaturation study. UV *T_m_* experiments suggest that FM1 CUG × 10 possesses a hairpin secondary structure. Initially, the self-folded hairpin secondary structure could hydrophobically embed the fluorescein and the overall fluorescence is minimized. Upon ligand binding at room temperature, the conformational change of the ligand-RNA complex gets the fluorophore exposed to the solution gradually along the binding process, leading to an observation of a binding curve based on the fluorescence readings. At elevated temperatures, along with conformational change due to the sisomicin-RNA binding, the FAM containing RNA is expected to have some thermodynamic change such as thermal motion/rotation. This effect can come into play and make a significant impact on fluorescence intensity, which will in turn affect the data accuracy towards the sisomicin-RNA interaction study. We designed a fluorescence stability test for annealed FM1 CUG × 10 at 20–80 °C to assess the latter factor. The temperature was increased from 20 to 80 °C at an increment of 5 °C and each temperature was held for totally 10 min (2 min hold time before each fluorescence reading, quintuplicate scans at each temperature). As shown in Figure S4, at higher temperatures (70–80 °C), fluorescence intensity kept increasing during a 10 min hold time for each temperature, meaning that the annealed RNA was losing the FAM-containing hydrophobic structure gradually and FAM was getting more exposed due to molecular thermal motion/rotation.

**Figure 4:**
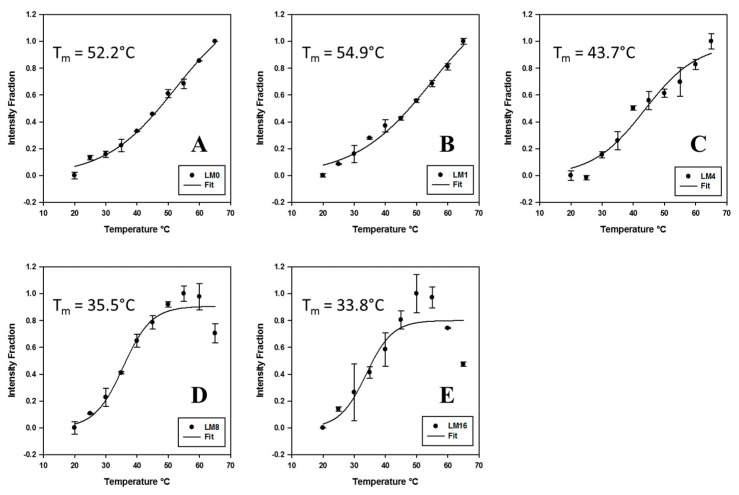
Sigmoidal fittings of *T_m_* curves for titrating sisomicin against annealed FM1 r(CUG)_10_: (**A**–**E**) *T_m_* at ligand to RNA molar ratios of 0, 1, 4, 8, and 16. Annealed FM1 r(CUG)_10_ RNA sample concentration 200 nM and volume size 150 µL; ligand addition volume size 1.5 µL; fluorescence setting: excitation at 485 nm, emission at 500–600 nm, and slit width of 3 nm in a range of 20–65 °C.

**Figure 5:**
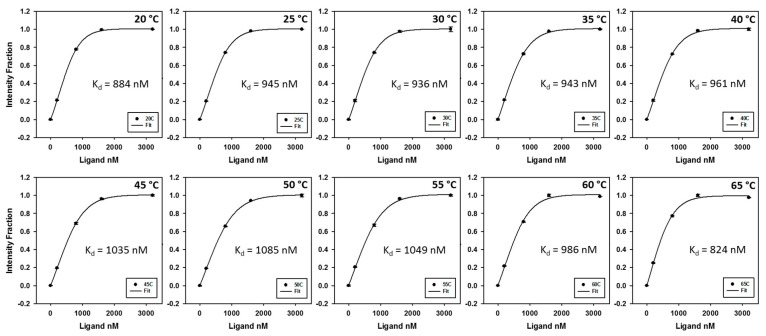
Sigmoidal fitting of sisomicin binding data and calculated K_d_ values at 20–65 °C. Annealed FM1 r(CUG)_10_ RNA sample concentration 200 nM and volume size 150 µL; sisomicin addition volume size 1.5 µL with ligand to RNA molar ratios of 0, 1, 4, 8, and 16; fluorescence setting: excitation at 485 nm, emission at 500–600 nm, and slit width of 3 nm.

**Figure 6:**
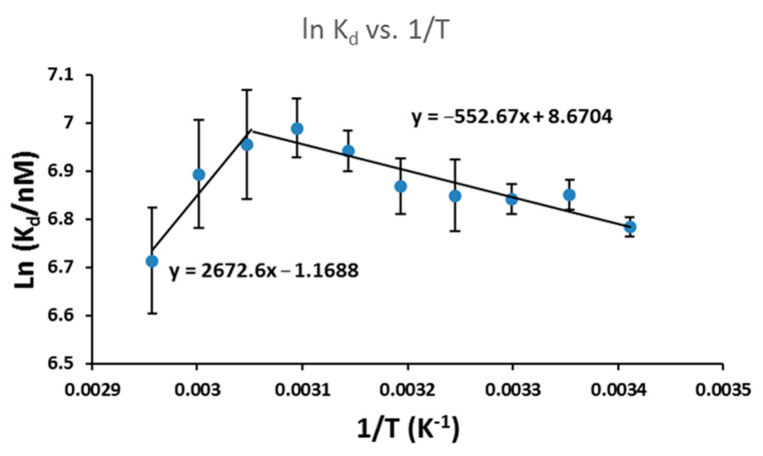
Van ’t Hoff plot for dissociation equilibrium of sisomicin-RNA repeats at 20–65 °C. Annealed FM1 r(CUG)_10_ RNA sample concentration 200 nM and volume size 150 µL; sisomicin addition volume size 1.5 µL with ligand to RNA molar ratios of 0, 1, 4, 8, and 16; fluorescence setting: excitation at 485 nm, emission at 500–600 nm, and slit width of 3 nm.

To probe more insights into the sisomicin-RNA interaction, we further carried out a thermal melting study employing fluorescence titration technique at 20–65 °C. Denatured FM1 r(CUG)_10_ RNA repeats were used as a control to be compared with annealed FM1 r(CUG)_10_ RNA repeats having hairpin secondary structures. Results with denatured and annealed RNA repeats are shown in Figure 4, Figure 5 and Figure 6 and Figures S5–S12, respectively. Normalized *T_m_* data points were plotted and *T_m_* values were calculated through sigmoidal fittings for annealed FM1 CUG × 10 in Figure 4. With no sisomicin addition, the plot in Figure 4A generates a calculated *T_m_* of 52.2 °C to be compared with a UV *T_m_* value of 55.8 °C in Table S2 (FM1 labeled as r10FAM in UV *T_m_* study). According to the results in Figure S4, the effect of thermal motion/rotation of the fluorescein is negligible below 65 °C. As a consequence, this fluorescence *T_m_* curve in Figure 4A reflects the conformation change of the annealed FM1 RNA structure during thermal denaturation at 20–65 °C. Our UV *T_m_* data in Table S2 show that ligand addition can stabilize the annealed RNA structure with a slight increase in *T_m_* value (an increase of ~1.5 °C with sisomicin addition initially at L/M = 1 and no change when continuing sisomicin addition to L/M = 4). Similarly, the fluorescence *T_m_* curve in Figure 4B gives a *T_m_* value of 54.9 °C with sisomicin addition at L/M = 1, increasing 2.7 °C from the initial value. However, the rest of the plots in Figure 4 show a significant decrease of *T_m_* with L/M > 1, contradicting the trend in UV *T_m_* experiments. To explain this inconsistency, we need to introduce a new parameter below to discuss the stability of annealed FM1 RNA structure.

In this study, the fluorescence intensity of RNA samples (either peak value or value at 520 nm of each fluorescence spectrum) generally increases with temperature under the experimental conditions. Here, we define ∆F as the maximum change of fluorescence intensity (basically the difference of fluorescence intensity values between 20 and 65 °C) in each graph in Figures S5 and S8. As discussed above, the fluorescence change caused by thermal motion/rotation of the fluorophore in annealed FM1 RNA is negligible below 65 °C. Accordingly, the fluorescence change ∆F is directly correlated to the conformation change of annealed FM1 RNA structure under the experimental conditions. Given an extreme example, if ∆F = 0, the RNA structure would be super stable with no conformation change at all when increasing temperature from 20 to 65 °C. Therefore, ∆F is an indicator/measurement for the stability of RNA structures. In other words, the smaller ∆F, the more stable the RNA structure. In the case of sisomicin addition, this still holds true assuming no significant thermal motion of sisomicin is involved below 65 °C. As ∆F is a subtraction of the lowest fluorescence value from the highest one at 20–65 °C, it is like a blank run subtraction and any small ligand interference/background would be removed. Thus, ∆F reflects the stability of the sisomicin-RNA complex in this case.

In Table 1, we calculated ∆F from fluorescence spectra in Figures S5 and S8 for denatured and annealed FM1 CUG × 10 RNA repeats titrated by sisomicin. Denatured and annealed RNA repeats behaved differently during thermal melting in the studied temperature range of 20–65 °C. Their distinctive patterns when binding with sisomicin reveal some structural information. For denatured CUG repeats, as they possess very few secondary structures, they would have a higher ∆F value. On the other hand, for annealed CUG repeats, since they have some secondary structures, their ∆F value would be smaller. Our data showed that with no sisomicin ligand added, ∆F for denatured CUG repeats was close to 1.5 fold higher than the one for annealed RNA repeats. When ligand binding is involved, the minimum ∆F suggests the most stable state of the ligand-RNA complex structure. Interestingly, the minimum ∆F occurs coincidentally when binding sites of RNA structure are being saturated, indicating that the most stable structure is the ligand-RNA complex with all binding sites being occupied.

As discussed in our UV optical melting study, binding with a ligand can enhance the stability of the RNA structure. Surprisingly, even denatured CUG repeats showed some binding with sisomicin, suggesting that the denatured repeat sequence still possessed some random structure—possibly a random loose coil with FAM being concealed from exposure. At an L/M ratio of 1, ∆F was minimized bearing a sharp change from the highest ∆F with no sisomicin added, implying that the denatured RNA structure only had approximately one site per RNA molecule. Increasing sisomicin concentration would increase some other interactions (e.g., non-specific binding, multi-layer adsorption, etc.) and make the sisomicin-RNA random coil structure get slightly less stable or less compact. However, ∆F remained at ~9000, showing that the structure did not change much with L/M > 1 and was more stable than the case with no sisomicin added. For annealed CUG repeats, the most stable state of ligand-RNA structure was reached at an L/M ratio of 4, which is consistent with the self-folded hairpin structure having approximately four or five binding sites (a hairpin loop and four stem-loops, however, the first stem-loop next to 5′ end may not be stable). Without ligand added, annealed CUG repeats exhibited higher stability with a smaller value of ∆F because they possessed some secondary structure in comparison with denatured CUG repeats. At the L/M ratio of 1, its stability did not change much as ∆F increased slightly. When the L/M ratio reached 4, the binding sites of the annealed RNA structure were saturated and ∆F was minimized, indicating under these conditions the complex of annealed CUG repeats binding with sisomicin reached the most stable state. When L/M > 4, the complex had some conformation change: FAM got slightly more exposed and the stability decreased from the most stable state to become close to the state when L/M = 1.

Results of ligand concentration effect on binding at different temperatures were plotted in Figures S6 and S11. For denatured RNA, shown in spectra at each temperature, the lowest fluorescence emission curve was recorded when L/M = 0, and right above it was the emission curve with L/M = 1. Additionally, then the maximum emission spectrum was obtained when increasing sisomicin concentration to L/M = 4. This suggests that the binding of denatured CUG repeats with sisomicin was saturated between L/M = 1 and 4. Based on our ∆F discussion, it is possible that there would be some additional interactions like non-specific binding and multi-layer adsorption after the single binding site per denatured RNA strand was occupied. A similar situation also occurred to the case of annealed CUG repeats: the lowest emission curve appeared at L/M = 0; when increasing sisomicin concentration, emission curves at L/M = 8 and 16 became overlapped and were the highest, which together with binding curves in Figure 5 indicates that the binding reaches to the saturated state at L/M = 4.

Shown in Figure 5 and Figure S12, K_d_ values were calculated using sigmoidal fitting. In the case of multiple binding sites (annealed CUG repeats), sigmoidal curve fitting worked very well. However, sigmoidal curves fit poorly in the single binding site case (denatured CUG repeats), suggesting possible non-specific binding and/or multilayer adsorption domination when ligand was added continuously beyond the single binding site saturation. As K_d_ is the dissociation equilibrium constant, thermodynamic parameters of the dissociation of sisomicin with annealed CUG repeats were calculated using the Van ’t Hoff equation to plot lnK_d_ vs. 1/T in Figure 6. Unexpectedly, the linear regression showed there were two regions under the experimental conditions: (1) low temperature region from 20 to 50 °C with a free energy of −16.8 kJ/mol (standard 25 °C); and (2) high temperature region from 55 to 65 °C with a free energy of −19.3 kJ/mol (standard 25 °C). Two trend lines cross at 54.5 °C, which gives a *T_m_* very close to those values at L/M = 0 and 1 in Figure 4 (52.2 and 54.9 °C, respectively) and is only ~1 °C less than UV-melting temperature of FM1 r(CUG)_10_ (in Table S2, for r10FAM at L/M = 0, *T_m_* = 55.8 °C). This provides a new method to determine *T_m_*. Moreover, the presence of two regions indicates that the dissociation of the RNA-ligand complex would become more energetically favorable beyond *T_m_*.

In return to the discussion of the *T_m_* inconsistency in Figure 4 compared to UV *T_m_* results, ∆F was minimum at L/M = 4, meaning that the sisomicin-RNA complex should achieve the most stable state with a higher calculated *T_m_* on the fluorescence *T_m_* plot. Below 65 °C, thermal motion/rotation of FAM is negligible for annealed CUG RNA repeats, so it is true that the sisomicin-RNA complex is most stable at L/M = 4 as ∆F is the minimum. To explain the *T_m_* disagreement, keeping in mind that the thermal melting is a dynamic process and *T_m_* is defined as the temperature at which half of the nucleotide strands are in the random coil or single-stranded state. At the melting temperature *T_m_*, FM1 CUG × 10 RNA has two states: annealed self-folded hairpin structure and random coil/single-stranded structure with a molar ratio of 1:1. At this point, although the denatured half nucleotide strands are in coil/single-stranded state, the hydrophobic structure of FAM in the sequence is preserved, that is, the exposure degree of FAM to the solution remains unchanged, which is supported by the data in Figure S4: in total 10 min hold time, quintuplicate spectra at a fixed temperature below 65 °C did not show any significant changes in fluorescence intensity. When applying the same cooling rate, these denatured nucleotide strands would be annealed back to the self-folded hairpin structure. With no sisomicin addition, the actual structures of the denatured nucleotide strands do not matter as long as the fluorescence intensity at each temperature data point below 65 °C is constant. The fluorescence *T_m_* curve in Figure 4A depicted the thermal melting process at a range of 20–65 °C and gave a calculated *T_m_* of 52.2 °C at L/M = 0. Sisomicin addition can stabilize the self-folded RNA hairpin structure. As previously discussed with our denatured CUG × 10 RNA data, the random coil/single-stranded structure can still have one binding site for small molecules. Hence, a calculated higher *T_m_* was achieved for the fluorescence *T_m_* curve in Figure 4B. With L/M = 4, things become different. Indicated by ∆F, the sisomicin-RNA complex with sisomicin to RNA molar ratio as 4:1 is the most stable state. When this compact structure becomes a coil/single-stranded one due to thermal denaturation at 20–65 °C, the interactions of sisomicin and coil/single-stranded RNA include not only binding but also non-specific binding and/or multilayer adsorption, which would reduce the exposure of FAM to the solution. As the contribution to the total fluorescence intensity from denatured RNA gets lesser, the fluorescence *T_m_* curve of the sisomicin-RNA complex becomes sigmoidal and gives a lower *T_m_*. Continuing to increase L/M, the folded RNA hairpin structure is already saturated, meanwhile, non-specific binding and/or multilayer adsorption on the coil/single-stranded RNA structure become dominant and significantly reduce the fluorescence intensity from these RNA structures, resulting in the overall fluorescence intensity on the fluorescence *T_m_* curves at L/M = 8 and 16 even started to decrease at 55 and 50 °C, respectively. The interaction and conformation changes of the sisomicin-RNA complex at *T_m_* explain the existence of two regions for the dissociation of the RNA-ligand complex and validate this new *T_m_* determination approach.

### 2.5. CD Titration and Temperature Study

**Figure 7:**
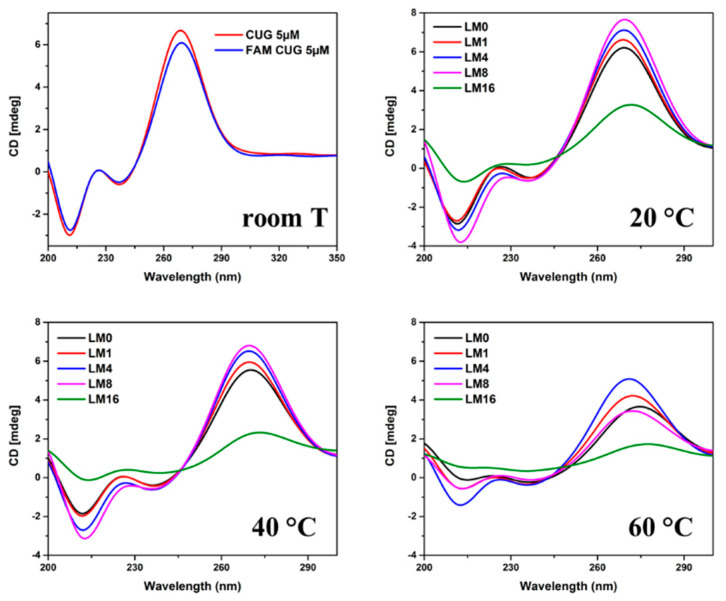
CD spectra of native r(CUG)_10_ as control at room temperature and FM1 r(CUG)_10_ as targeted RNA template for sisomicin titration at 20–60 °C. All annealed RNA samples were prepared at 5 µM with a volume size of 200 µL; sisomicin to RNA molar ratios of 0, 1, 4, 8, and 16; native r(CUG)_10_ CD spectrum from 350–200 nm and FM1 r(CUG)_10_ RNA–sisomicin CD spectra from 300–200 nm.

Conventional UV *T_m_* provides *T_m_* value as a good thermal stability indicator and other useful information such as thermodynamic parameters; however, it is not very sensitive at probing RNA-ligand interactions. Our fluorescence thermal melting assay, on the other hand, can provide all this information, and it is also very sensitive for studying RNA-ligand binding. By introducing a new parameter ∆F, it can depict the dynamic binding process and point out that the most stable state for a RNA-ligand complex is when all its binding sites are just occupied. Although our fluorescence experimental data back up this statement, unfortunately it only stands as a reasonable proposal if we do not have substantial structural information to support it. Usually, simulation results can work as useful structural supporting information to fulfill this assignment, however we would like to solve this issue from an experimental basis if possible. Circular dichroism (CD), as the best known technique to measure the effects of polarized light on chiral biomolecules such as nucleic acids, can give detailed structural and enantiomeric insights on both small molecule and macromolecular structures and has been widely used to provide profound secondary structure information on nucleotides [46]. We used native CUG × 10 RNA as a control and performed a CD temperature study with sisomicin titrating targeted FM1 CUG × 10 RNA template. As shown in Figure 7, our CUG × 10 RNA samples exhibited typical A-form secondary structures [46,47,48]. We used FM1 CUG × 10 RNA as the template to be consistent with our fluorescence thermal melting studies, and first investigated whether the mutation of incorporating FAM into the hairpin loop can cause any perturbation on the A-form secondary structure of CUG × 10 RNA or not. The upper left CD spectra in Figure 7 show that there were negligible changes between FM1 CUG × 10 RNA and the control of native CUG × 10 RNA, indicating that our FAM modified RNA probe conserved the original secondary structure and this fluorescence label design would not disturb the RNA-ligand binding as long as FAM has no direct interaction with ligands. Interestingly, sisomicin binding spectra at each temperature showed a different pattern from what was shown in Figure 3B,C. Generally the CD spectra in Figure 7 support that sisomicin can stabilize the folded CUG RNA structure. Initially, enhanced A-form secondary structure signals were observed with increasing sisomicin concentration up to L/M = 4 at each temperature. Continuing the sisomicin addition, changes on the A-form signals became temperature dependent (all A-form ellipticity signals discussed later in this article are referred to the positive peak near 270 nm in CD spectra).

**Figure 8:**
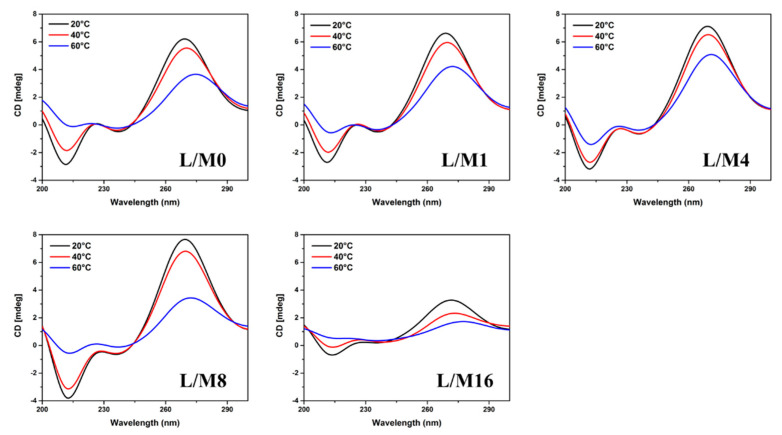
CD spectra of temperature effect on sisomicin binding towards annealed RNA repeats with different L/M. Annealed FM1 r(CUG)_10_ RNA sample at 5 µM with a volume size of 200 µL; sisomicin to RNA molar ratios of 0, 1, 4, 8, and 16; FM1 r(CUG)_10_ RNA–sisomicin CD spectra from 300–200 nm at 20–60 °C.

Traditionally, thermal melting of hairpin nucleotides is considered having only two states: folded and unfolded. However, the existence of multiple states has been suggested and observed in some literature [49,50,51], e.g., a transition state with the formation of intermediate collapsed structures (only end-to-end contact). As shown in Figure 7, the sisomicin binding with L/M = 8 had the highest intensity of A-form signal (270 nm) at 20 °C, but at 40 °C, signal intensity decreased to the same level as the one with L/M = 4, and at elevated temperature of 60 °C, it became even lower than the one with L/M = 0. More surprisingly, continuing the sisomicin titration to L/M = 16, a significant loss of A-form signal was recorded even at 20 °C. These observations suggest the existence of an intermediate state between folded and unfolded states: after all binding sites of the folded RNA molecule are occupied, continuing the sisomicin addition can eventually break up the closing GC base pair at the hairpin loop side, meanwhile the 5′ end GC base pair is still intact, leading to an intermediate structure with a larger size of hairpin loop and a shorter hairpin stem, which can accommodate more ligand molecules for binding. This is implied by the CD spectra along the binding process at each fixed temperature: at L/M = 16, CD spectra still have the signature signals of A-form secondary structures, but at a significantly lower intensity level, indicating shorter hairpin stems. We further examined the temperature effect on RNA-sisomicin binding with CD spectra in Figure 8. We applied the same strategy as our previously used one in the fluorescence thermal melting assay. Typically, ellipticity decreases as temperature increases. Here, we define ∆e as the maximum change of ellipticity at a specific temperature range in each graph in Figure 8. Calculated ∆e values are listed in Table 2. Except that at L/M = 16 ∆e values are not valid due to low ellipticity signals, ∆e works as a stability indicator just like ∆F: the smaller ∆e, the more stable the folded RNA structure. This gives us the most stable state at L/M = 4 in agreement with our results from fluorescence thermal melting studies.

## 3. Discussion

The biggest challenge on thermal stability studies of RNA-ligand interactions using fluorescence spectroscopy is how to differentiate fluorescence conformational changes due to ligand binding from those caused by thermal melting. We introduced a new parameter of ∆F to overcome this obstacle, showing that ∆F can work as a stability indicator and reach its minimum when all RNA binding sites are occupied by ligands. Then, we used CD as a complementary technique providing structural supporting information and verified this finding. In conclusion, we have reported two sensitive and efficient fluorescence assays based on a novel fluorescence-labeled CUG RNA sequence designed to address two major tasks/challenges in drug discovery towards pathogenic CUG RNA repeats. The microplate assay can be easily developed into a high-throughput screening assay for identifying lead ligands targeting disease-causing RNA expansions, offering both convenience and flexibility in terms of HTS and binding characterization. Consistent with the classic UV *T_m_* technique, the fluorescence thermal melting assay provides a new approach to determine *T*_m_ with extensive binding characterization such as probing thermal stability and measuring thermodynamic parameters, binding constants, and kinetics. Our assays can also be useful for the possible structural optimization of lead compound’s derivatives through differentiating fluorescence changes caused by adding/modifying some functional groups in the compound structure. This combined with our future structural and simulation studies would have great potential to speed up the drug discovery process.

## 4. Materials and Methods

Native and FAM-modified RNA oligonucleotides in Table S1 were chemically synthesized by solid-phase synthesis using a BIOSSET ASM-800 Oligo synthesizer. Solutions of ligands and RNAs were all prepared in AMBION nuclease-free water. Stock solutions of ligands were prepared from commercially available Sisomicin Sulfate, Kanamycin Sulfate, and Neomycin trisulfate hydrate salts, purchased from Krackeler scientific (Albany, NY, USA) and Fisher scientific (Waltham, MA, USA).

### 4.1. Fluorescence Microplate Assay

The FAM modified RNA repeated sequence was used as an RNA template for the microplate assay. The aminoglycoside comparison experiment was carried out on 96-well plates using a BioTek^®^ Synergy H1 hybrid multi-mode reader as the fluorescence readout operated with settings as follows: excitation at 485 nm; emission at 520 nm; and gain at 100. The RNA template employed in the assay was prepared by heating FAM modified r(CUG)_10_ RNA repeats at 95 °C for 5 min, followed by slow cooling to room temperature for ~2 h. Wells loaded with 100 µL of 200 nM annealed RNA repeats were subject to a 1-µL ligand addition in different concentrations (10, 20, 40, 80, 160, and 320 µM) generating ligand to RNA molar ratios—L/M at 0.5, 1, 2, 4, 8, and 16. The RNA sample plates were scanned by the microplate reader before and after ligand addition to calculate fluorescence changes. Experiments were performed in triplicate with three accumulations. Binding modeling with sigmoidal data fitting (Figure S1) was processed using SigmaPlot software.

### 4.2. Fluorescence Thermal Melting Assay

Fluorescence thermal denaturation experiments were carried out on a Fluorolog-3-22 spectrofluorometer from HORIBA Jobin Yvon equipped with an LFI-3751 temperature controller. A typical 150 µL volume of 200 nM RNA sample in either denatured (95 °C for 5 min, then on ice) or annealed (95 °C for 5 min, then slowly cooling to room temperature for ~2 h) form was loaded in a 200 µL cuvette and subsequently titrated by addition of 1.5 µL ligand in different concentrations (generating 0 to 3200 nM ligand in solution or ligand to RNA molar ratios—L/M at 0, 1, 4, 8, and 16). Each concentration of the mixture was studied by heating and cooling samples at a range of 20 to 65 °C with an increment of 5 °C and a 2 min hold time at each temperature. Excited at 485 nm, fluorescence emission spectra were recorded in a range of 500–600 nm with a 3 nm slit width. Raw spectra were smoothed with the Lowess 11 pt method and fluorescence readings at the wavelength of 520 nm were used for data analysis to be consistent with our fluorescence microplate assay. Experiments were carried out at least in triplicate. Data fitting was performed with SigmaPlot software.

### 4.3. Circular Dichroism (CD) Experiments

CD experiments were performed on a Jasco-815 CD spectrometer at either room temperature or specific temperatures utilizing a Peltier temperature controller. A typical 5 µM RNA sample annealed in nuclease-free water (95 °C for 5 min, then cooled down slowly to room temperature for ~2 h, and incubated at 4 °C for at least 2 h) was prepared in 200 µL volume and loaded in a 350 µL quartz cell with a 1 mm path length. In a heated CD titration experiment, a prepared RNA sample was subsequently titrated by equivalent sisomicin to generate ligand to RNA molar ratios—L/M at 0, 1, 4, 8, and 16. Then, each concentration of the mixture was studied at 20, 40, and 60 °C, respectively, by heating and cooling the sample at 1 °C/min with a 2 min hold time for each temperature. CD spectra were collected at a scanning speed of 100 nm/min from the wavelength of 300 to 200 nm and with 1.0 nm bandwidth and 1.0 s digital integration time. All raw CD spectra were smoothed with the Savitzky–Golay 25 pt method. There was no baseline correction needed for samples prepared in nuclease-free water, and experiments were carried out at least in triplicate.

## Figures and Tables

**Table 1:** Change in fluorescence ∆F (between 20 and 65 °C) for denatured and annealed FM1 CUG × 10.

L/M Ratio	Denatured RNA, ∆F	Annealed RNA, ∆F
0	14,700	11,000
1	7300	12,300
4	8200	8400
8	9000	13,200
16	10,400	14,200

**Table 2:** Change in ellipticity ∆e at 270 nm (between 20 and 60 °C) for annealed FM1 CUG × 10.

L/M Ratio	20–40 °C ∆e	20–60 °C ∆e	40–60 °C ∆e
0	0.66	2.55	1.89
1	0.67	2.41	1.74
4	0.60	2.03	1.43
8	0.85	4.22	3.36
16	0.95	1.55	0.60

## Data Availability

The data presented in this study are available in insert article or Supplementary Material.

## Supplementary Materials

The following supporting information can be downloaded at: https://www.mdpi.com/article/10.3390/ijms23063321/s1.

## Abbreviations

6-FAM	6-carboxyfluorescein
HIV-1	human immunodefiency virus type 1
TAR	trans-activation response element
DM1 or 2	myotonic dystrophy type 1 or 2
ALS	amyotrophic lateral sclerosis
HD	Huntington’s disease
FXTAS	fragile X-associated tremor ataxia syndrome
SCA8	spinocerebellar ataxia type 8
HDL2	Huntington’s disease-like 2
ASO	antisense oligonucleotide
HTS	high-throughput screening
UV *T_m_*	ultraviolet thermal melting
CD	circular dichroism
FRET	fluorescence resonance energy transfer
FID	fluorescent indicator displacement
MB	molecular beacon
K_d_	dissociation constant
E_λmax_	extinction coefficient

## References

[1] Cooper T.A., Wan L., Dreyfuss G. (2009). RNA and disease. Cell.

[2] Thornton C.A. (2014). Myotonic Dystrophy. Neurol. Clin..

[3] Rademakers R., Neumann M., Mackenzie I.R. (2012). Advances in understanding the molecular basis of frontotemporal dementia. Nat. Rev. Neurol..

[4] The Huntington’s Disease Collaborative Research Group (1993). A novel gene containing a trinucleotide repeat that is expanded and unstable on Huntington’s disease chromosomes. Cell.

[5] Orr H.T., Zoghbi H.Y. (2007). Trinucleotide repeat disorders. Annu. Rev. Neurosci..

[6] Ranum L.P., Cooper T.A. (2006). RNA-mediated neuromuscular disorders. Annu. Rev. Neurosci..

[7] O’Rourke J.R., Swanson M.S. (2009). Mechanisms of RNA-mediated disease. J. Biol. Chem..

[8] Timchenko L.T., Miller J.W., Timchenko N.A., DeVore D.R., Datar K.V., Lin L., Roberts R., Caskey C.T., Swanson M.S. (1996). Identification of a (CUG)n triplet repeat RNA-binding protein and its expression in myotonic dystrophy. Nucleic Acids Res..

[9] Wang J., Pegoraro E., Menegazzo E., Gennarelli M., Hoop R.C., Angelini C., Hoffman E.P. (1995). Myotonic dystrophy: Evidence for a possible dominant-negative RNA mutation. Hum. Mol. Genet..

[10] Barreau C., Paillard L., Mereau A., Osborne H.B. (2006). Mammalian CELF/Bruno-like RNA-binding proteins: Molecular characteristics and biological functions. Biochimie.

[11] Pascual M., Vicente M., Monferrer L., Artero R. (2006). The Muscleblind family of proteins: An emerging class of regulators of developmentally programmed alternative splicing. Differ. Res. Biol. Divers..

[12] Sazani P., Kole R. (2003). Therapeutic potential of antisense oligonucleotides as modulators of alternative splicing. J. Clin. Investig..

[13] Wheeler T.M., Sobczak K., Lueck J.D., Osborne R.J., Lin X., Dirksen R.T., Thornton C.A. (2009). Reversal of RNA dominance by displacement of protein sequestered on triplet repeat RNA. Science.

[14] Mulders S.A., van den Broek W.J., Wheeler T.M., Croes H.J., van Kuik-Romeijn P., de Kimpe S.J., Furling D., Platenburg G.J., Gourdon G., Thornton C.A. (2009). Triplet-repeat oligonucleotide-mediated reversal of RNA toxicity in myotonic dystrophy. Proc. Natl. Acad. Sci. USA.

[15] Boudreau R.L., Martins I., Davidson B.L. (2009). Artificial microRNAs as siRNA shuttles: Improved safety as compared to shRNAs in vitro and in vivo. Mol. Ther. J. Am. Soc. Gene Ther..

[16] Arambula J.F., Ramisetty S.R., Baranger A.M., Zimmerman S.C. (2009). A simple ligand that selectively targets CUG trinucleotide repeats and inhibits MBNL protein binding. Proc. Natl. Acad. Sci. USA.

[17] Warf M.B., Nakamori M., Matthys C.M., Thornton C.A., Berglund J.A. (2009). Pentamidine reverses the splicing defects associated with myotonic dystrophy. Proc. Natl. Acad. Sci. USA.

[18] Pushechnikov A., Lee M.M., Childs-Disney J.L., Sobczak K., French J.M., Thornton C.A., Disney M.D. (2009). Rational design of ligands targeting triplet repeating transcripts that cause RNA dominant disease: Application to myotonic muscular dystrophy type 1 and spinocerebellar ataxia type 3. J. Am. Chem. Soc..

[19] Lee M.M., Pushechnikov A., Disney M.D. (2009). Rational and modular design of potent ligands targeting the RNA that causes myotonic dystrophy 2. ACS Chem. Biol..

[20] Garcia-Lopez A., Monferrer L., Garcia-Alcover I., Vicente-Crespo M., Alvarez-Abril M.C., Artero R.D. (2008). Genetic and chemical modifiers of a CUG toxicity model in Drosophila. PLoS ONE.

[21] Childs-Disney J.L., Yildirim I., Park H., Lohman J.R., Guan L., Tran T., Sarkar P., Schatz G.C., Disney M.D. (2014). Structure of the myotonic dystrophy type 2 RNA and designed small molecules that reduce toxicity. ACS Chem. Biol..

[22] Disney M.D., Yildirim I., Childs-Disney J.L. (2014). Methods to enable the design of bioactive small molecules targeting RNA. Org. Biomol. Chem..

[23] Hoskins J.W., Ofori L.O., Chen C.Z., Kumar A., Sobczak K., Nakamori M., Southall N., Patnaik S., Marugan J.J., Zheng W. (2014). Lomofungin and dilomofungin: Inhibitors of MBNL1-CUG RNA binding with distinct cellular effects. Nucleic Acids Res..

[24] Luo Y., Disney M.D. (2014). Bottom-up Design of Small Molecules that Stimulate Exon 10 Skipping in Mutant MAPT Pre-mRNA. Chembiochem. A Eur. J. Chem. Biol..

[25] Rzuczek S.G., Park H., Disney M.D. (2014). A toxic RNA catalyzes the in cellulo synthesis of its own inhibitor. Angew. Chem. Int. Ed..

[26] Su Z., Zhang Y., Gendron T.F., Bauer P.O., Chew J., Yang W.Y., Fostvedt E., Jansen-West K., Belzil V.V., Desaro P. (2014). Discovery of a Biomarker and Lead Small Molecules to Target r(GGGGCC)-Associated Defects in c9FTD/ALS. Neuron.

[27] Tran T., Childs-Disney J.L., Liu B., Guan L., Rzuczek S., Disney M.D. (2014). Targeting the r(CGG) repeats that cause FXTAS with modularly assembled small molecules and oligonucleotides. ACS Chem. Biol..

[28] Velagapudi S.P., Disney M.D. (2014). Two-dimensional combinatorial screening enables the bottom-up design of a microRNA-10b inhibitor. Chem. Commun. (Camb.).

[29] Velagapudi S.P., Gallo S.M., Disney M.D. (2014). Sequence-based design of bioactive small molecules that target precursor microRNAs. Nat. Chem. Biol..

[30] Wong C.H., Nguyen L., Peh J., Luu L.M., Sanchez J.S., Richardson S.L., Tuccinardi T., Tsoi H., Chan W.Y., Chan H.Y. (2014). Targeting toxic RNAs that cause myotonic dystrophy type 1 (DM1) with a bisamidinium inhibitor. J. Am. Chem. Soc..

[31] Stelzer A.C., Frank A.T., Kratz J.D., Swanson M.D., Gonzalez-Hernandez M.J., Lee J., Andricioaei I., Markovitz D.M., l-Hashimi H.M. (2011). Discovery of selective bioactive small molecules by targeting an RNA dynamic ensemble. Nat. Chem. Biol..

[32] Parkesh R., Childs-Disney J.L., Nakamori M., Kumar A., Wang E., Wang T., Hoskins J., Tran T., Housman D., Thornton C.A. (2012). Design of a bioactive small molecule that targets the myotonic dystrophy type 1 RNA via an RNA motif-ligand database and chemical similarity searching. J. Am. Chem. Soc..

[33] Kumar A., Parkesh R., Sznajder L.J., Childs-Disney J.L., Sobczak K., Disney M.D. (2012). Chemical correction of pre-mRNA splicing defects associated with sequestration of muscleblind-like 1 protein by expanded r(CAG)-containing transcripts. ACS Chem. Biol..

[34] Disney M.D., Liu B., Yang W.Y., Sellier C., Tran T., Charlet-Berguerand N., Childs-Disney J.L. (2012). A small molecule that targets r(CGG) (exp) and improves defects in fragile X-associated tremor ataxia syndrome. ACS Chem. Biol..

[35] Yang W.Y., Gao R., Southern M., Sarkar P.S., Disney M.D. (2016). Design of a bioactive small molecule that targets r(AUUCU) repeats in spinocerebellar ataxia 10. Nat. Commun..

[36] Wicks S.L., Hargrove A.E. (2019). Fluorescent indicator displacement assays to identify and characterize small molecule interactions with RNA. Methods.

[37] Garner A.L. (2018). cat-ELCCA: Catalyzing drug discovery through click chemistry. Chem. Commun. (Camb.).

[38] Liu B., Diamond J.M., Mathews D.H., Turner D.H. (2011). Fluorescence competition and optical melting measurements of RNA three-way multibranch loops provide a revised model for thermodynamic parameters. Biochemistry.

[39] Gluszynska A., Juskowiak B., Rubis B. (2018). Binding Study of the Fluorescent Carbazole Derivative with Human Telomeric G-Quadruplexes. Molecules.

[40] Cardullo R.A., Agrawal S., Flores C., Zamecnik P.C., Wolf D.E. (1988). Detection of nucleic acid hybridization by nonradiative fluorescence resonance energy transfer. Proc. Natl. Acad. Sci. USA.

[41] Jiao Y., Stringfellow S., Yu H. (2002). Distinguishing “looped-out” and “stacked-in” DNA bulge conformation using fluorescent 2-aminopurine replacing a purine base. J. Biomol. Struct. Dyn..

[42] Bonnet G., Tyagi S., Libchaber A., Kramer F.R. (1999). Thermodynamic basis of the enhanced specificity of structured DNA probes. Proc. Natl. Acad. Sci. USA.

[43] Tsourkas A., Behlke M.A., Rose S.D., Bao G. (2003). Hybridization kinetics and thermodynamics of molecular beacons. Nucleic Acids Res..

[44] You Y., Tataurov A.V., Owczarzy R. (2011). Measuring thermodynamic details of DNA hybridization using fluorescence. Biopolymers.

[45] Mukherjee S., Blaszczyk L., Rypniewski W., Falschlunger C., Micura R., Murata A., Dohno C., Nakatani K., Kiliszek A. (2019). Structural insights into synthetic ligands targeting A–A pairs in disease-related CAG RNA repeats. Nucleic Acids Res..

[46] Ranjbar B., Gill P. (2009). Circular dichroism techniques: Biomolecular and nanostructural analyses—A review. Chem. Biol. Drug Des..

[47] Meroueh M., Chow C.S. (1999). Thermodynamics of RNA hairpins containing single internal mismatches. Nucleic Acids Res..

[48] Hannoush R.N., Damha M.J. (2001). Remarkable stability of hairpins containing 2’,5’-linked RNA loops. J. Am. Chem. Soc..

[49] Ma H., Proctor D.J., Kierzek E., Kierzek R., Bevilacqua P.C., Gruebele M. (2006). Exploring the energy landscape of a small RNA hairpin. J. Am. Chem. Soc..

[50] Ma H., Wan C., Wu A., Zewail A.H. (2007). DNA folding and melting observed in real time redefine the energy landscape. Proc. Natl. Acad. Sci. USA.

[51] Bowman G.R., Huang X., Yao Y., Sun J., Carlsson G., Guibas L.J., Pande V.S. (2008). Structural insight into RNA hairpin folding intermediates. J. Am. Chem. Soc..

