# Supplementary material for: Fluorescence-Based Binding Characterization of Small Molecule Ligands Targeting CUG RNA Repeats
Source: Int J Mol Sci. 2022 Mar 19;23(6):3321. doi: 10.3390/ijms23063321 (PMC8955525; doi:10.3390/ijms23063321)
Supplement: Supplementary file 1 [file ijms-23-03321-s001.zip › ijms-1598000-supplementary.pdf]

Supporting Information

Fluorescence-based Binding Characterization of Small Molecule Ligands Targeting CUG RNA Repeats

Zhihua Chang [1], Yaying Zheng [1], Johnsi Mathivanan [1], Vibhav A. Valsangkar [1], Jinxi Du [1], Reham A. I. Abou-Elkhair [2], Abdalla E. A. Hassan [2,*] and Jia Sheng [1,*]

[1] Department of Chemistry and The RNA Institute, University at Albany, State University of New York, 1400 Washington Avenue, Albany, NY 12222, USA; zchang@albany.edu (Z.C.); yzheng21@albany.edu (Y.Z.); jmathivanan@albany.edu (J.M.); vibhavvalsangkar@gmail.com (V.A.V.); jdu4@albany.edu (J.D.)

[2] Applied Nucleic Acids Research Center & Chemistry Department, Faculty of Science, Zagazig University; riham31@yahoo.com

* Correspondence: habdallaa@aol.com (A.E.A.H.); jsheng@albany.edu (J.S.)

Table of Contents

Part I. Aminoglycoside comparison data and RNA sequences S2

Part II. UV-melting temperature (Tm) study of RNA CUG repeats S3

Part III. Fluorescence characterization on annealed CUG RNA repeats S5

Part IV. Fluorescence characterization on denatured CUG RNA repeats S7

Part I. Aminoglycoside comparison data and RNA sequences

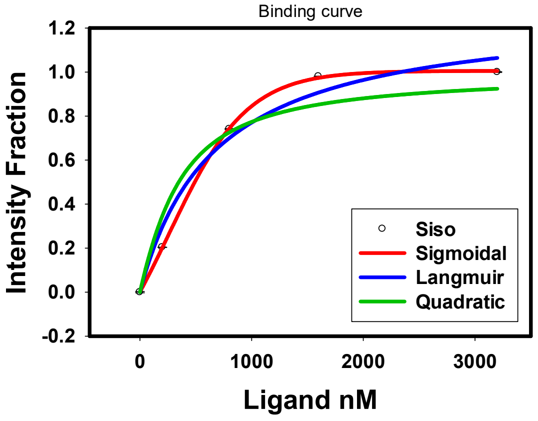

Figure S1. Comparison of different binding models with fluorometer data obtained at room temperature. Sigmoidal fitting (in red): K_d = 945 nM, R^2 = 0.9999. Modified Langmuir isothermal model regression (in blue): K_d = 671 nM, R^2 = 0.9766. Quadratic model fitting (in green): K_d = 247 nM, R^2 = 0.9493.

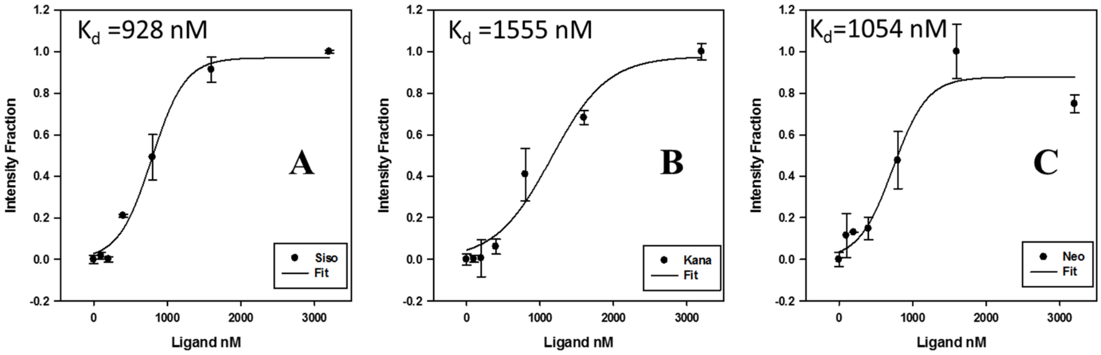

Figure S2. Sigmoidal fitting of aminoglycoside binding data and calculated K_d values. (A) Sisomicin: K_d = 928 nM, R^2 = 0.9898; (B) Kanamycin: K_d = 1555 nM, R^2 = 0.9639; (C) Neomycin: K_d = 1054 nM, R^2 = 0.9471.

Table S1. CUG repeats sequences.

Entry	Sequence
Native r7	5'-CUGCUGCUGCUGCUGCUGCUG-3'
Native r10	5'-CUGCUGCUGCUGCUGCUGCUGCUGCUGCUG-3'
FM1	5'-CUGCUGCUGCUGCfamUGCUGCUGCUGCUGCUG-3'

Part II. UV-melting temperature (T_m) study of RNA CUG repeats

Solutions of RNAs (1.5 μM) were prepared in sodium phosphate buffer (10 mM, pH 7.0) containing 100 mM NaCl. The samples were heated to 95 °C for 5 min, then cooled down slowly to room temperature, and incubated at 4 °C for at least 2 h before T_m measurements. Thermal denaturation was performed on a Cary 300 UV-Visible Spectrophotometer utilizing a temperature controller. The block temperature was the temperature reported. Denaturing curves were acquired at 260 nm with 4 ramps by heating and cooling samples from 5 to 85 °C at a rate of 0.5 °C/min. UV melting of binding studies was conducted with RNA samples subsequently titrated by ligands generating ratios of ligand to RNA from 0 to 16. The thermodynamic parameters of samples were obtained by fitting their melting curves in the MeltWin 3.5 software.

Table S2. Calculated T_m for Siso-CUG binding with Meltwin 3.5.

CUG Repeats	LM0	LM1	LM4	LM4_2	LM8	LM16
**r7**	59.8 ± 0.5°C	64.0 ± 1.3°C	61.9 ± 0.5°C	60.0 ± 0.3°C	62.4 ± 1.4°C	62.8 ± 1.0°C
**r10**	57.5 ± 0.5°C	58.7 ± 0.5°C	58.8 ± 0.2°C	59.4 ± 0.3°C	60.4 ± 0.5°C	60.7 ± 0.5°C
**r10FAM**	55.8 ± 0.6°C	57.4 ± 0.2°C	57.2 ± 0.4°C	57.2 ± 0.6°C	58.7 ± 0.5°C	58.6 ± 0.5°C

**r10FAM5µM**	57.1 ± 0.5°C	57.3 ± 0.7°C	59.0 ± 1.1°C	59.0 ± 0.7°C	59.6 ± 0.6°C	61.2 ± 1.0°C

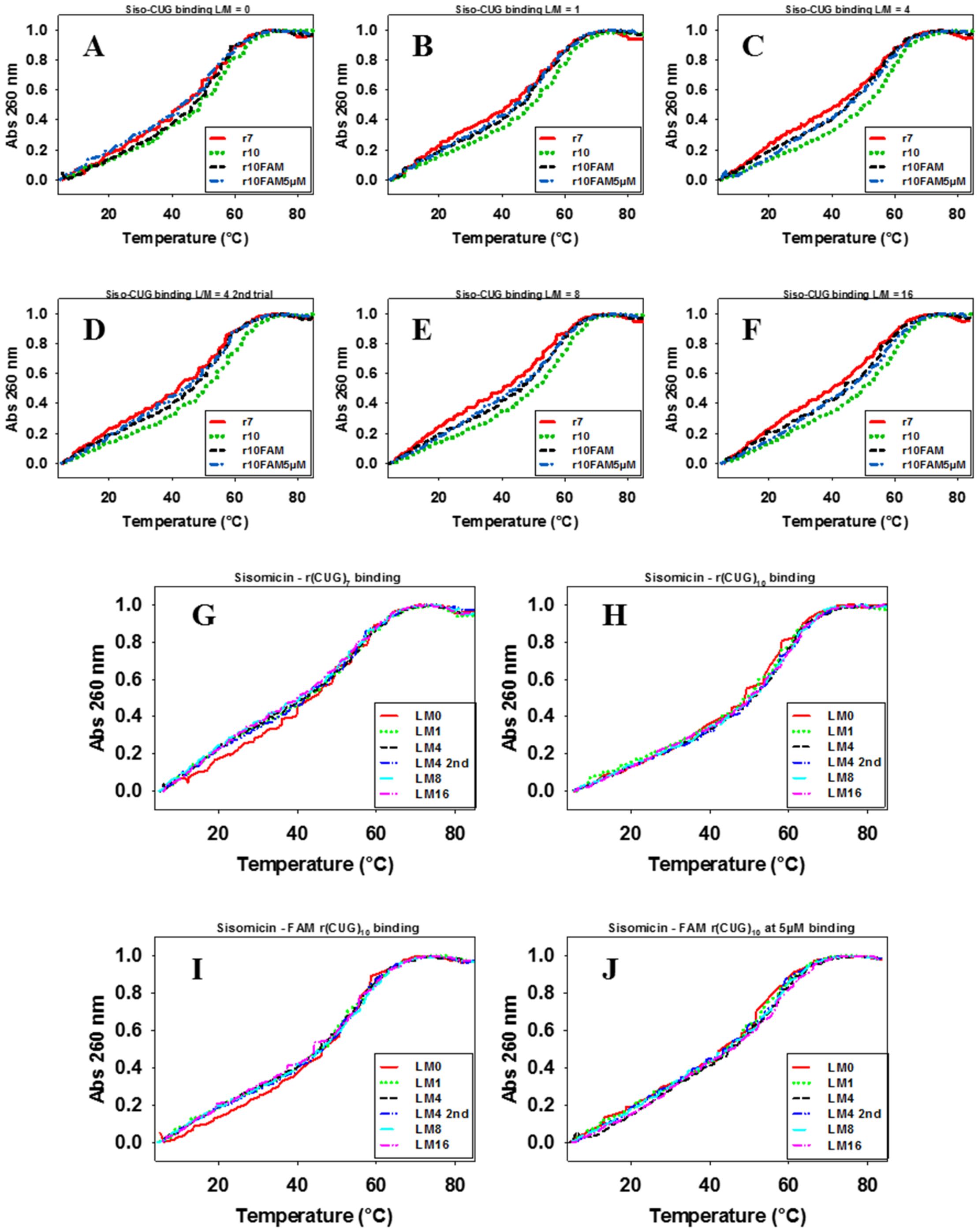

Figure S3. Normalized UV-melting curves of RNA-ligand complexes using sisomicin as titrant against different RNA repeat templates: r7 – native r(CUG)$_{10}$, r10 – native r(CUG)$_{10}$, r10FAM – FM1 r(CUG)$_{10}$, all at 1.5 µM, and r10FAM5µM – FM1 r(CUG)$_{10}$ at 5 µM. (A – F) T_m curves at equivalent ligand/RNA molar ratios of 0, 1, 4, 4, 8, and

16 respectively; LM4 2nd: a repeat of LM4 after volume loss (~10%) compensated by adding DI water. (G – J) Ligand concentration effect on different RNA repeat templates: r7, r10, r10FAM, and r10FAM5μM respectively. UV-melting running conditions: 600 μL annealed sample in sodium phosphate buffer (10 mM, pH 7.0) containing 100 mM NaCl; Absorbance recorded at 260 nm with 4 ramps in 5 – 85 °C (heating/cooling, 0.5 °C/min); T_m calculated with Meltwin 3.5 software.

Part III. Fluorescence characterization on annealed CUG RNA repeats

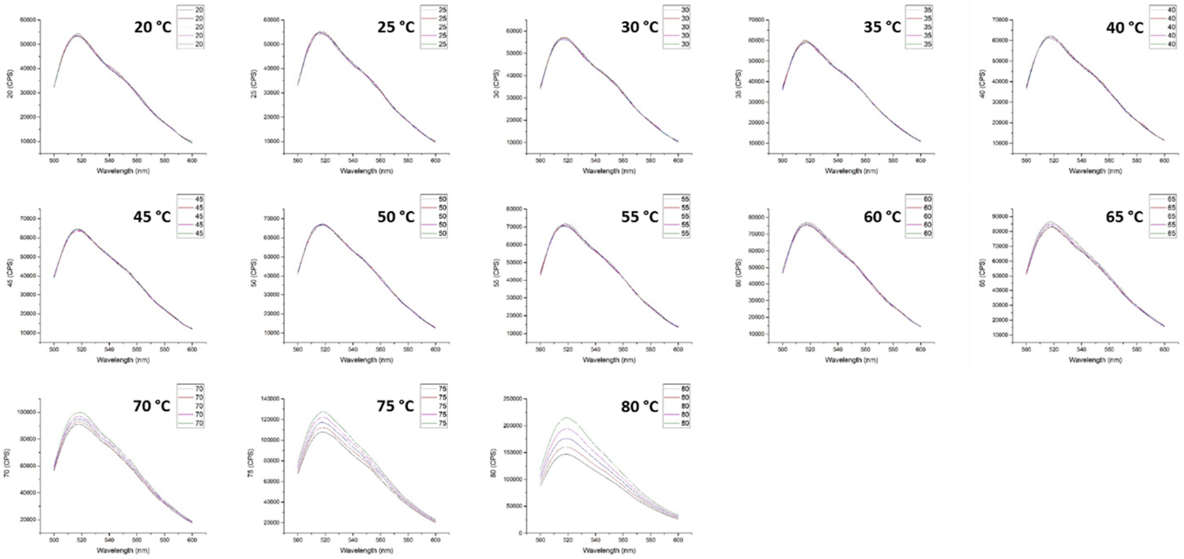

Figure S4. Quintuplicate spectra at each temperature of 20 – 80 °C for annealed RNA repeats. Hold time: 2 min for each scan, totally 10 min at each temperature; 150 μL of 200 nM annealed FM1 r(CUG)$_{10}$ sample with no sisomicin addition; Fluorescence setting: excitation at 485 nm, emission at 500 – 600 nm, and slit width of 3 nm.

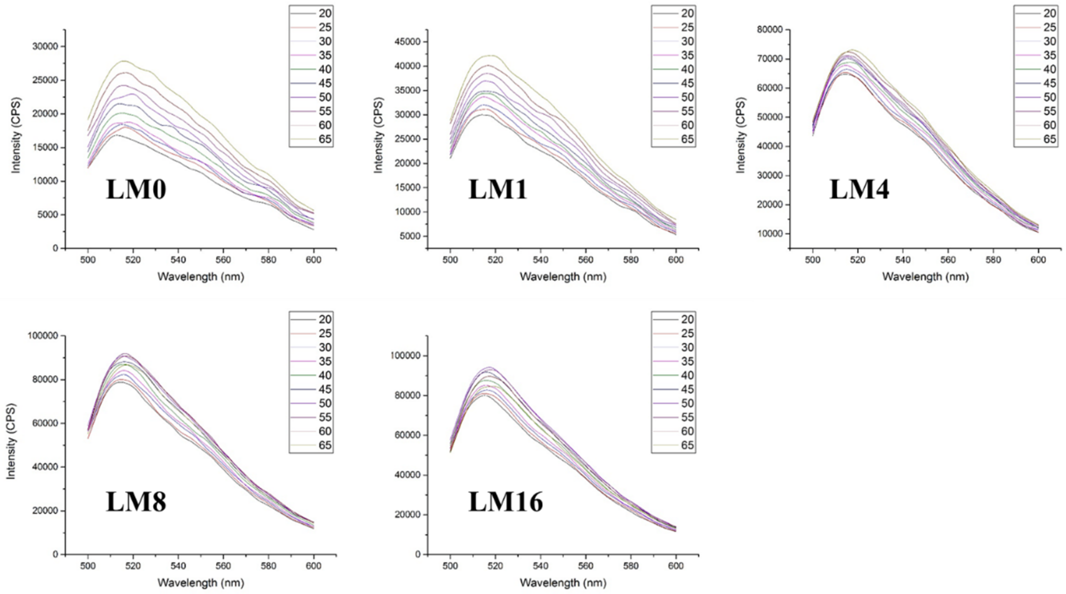

Figure S5. Fluorescence spectra of temperature effect on sisomicin binding towards annealed RNA repeats with different L/M. Annealed FM1 r(CUG)$_{10}$ RNA sample concentration 200 nM and volume size 150 µL; Sisomicin addition volume size 1.5 µL with ligand to RNA molar ratios – L/M at 0, 1, 4, 8, and 16; Fluorescence setting: excitation at 485 nm, emission at 500 – 600 nm, and slit width of 3 nm at 20 – 65 °C.

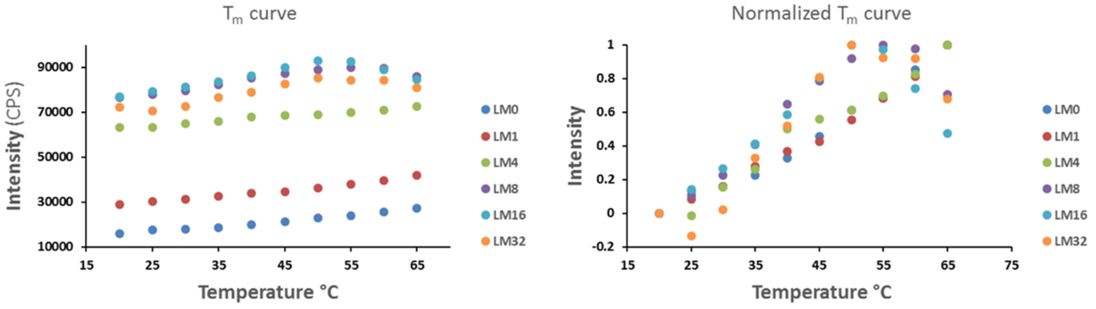

Figure S6. T_m curves for sisomicin binding towards annealed RNA with different L/M. Annealed FM1 r(CUG)$_{10}$ RNA sample concentration 200 nM and volume size 150 µL; Sisomicin addition volume size 1.5 µL with ligand to RNA molar ratios – L/M at 0, 1, 4, 8, and 16; Fluorescence setting: excitation at 485 nm, emission at 500 – 600 nm, and slit width of 3 nm at 20 – 65 °C.

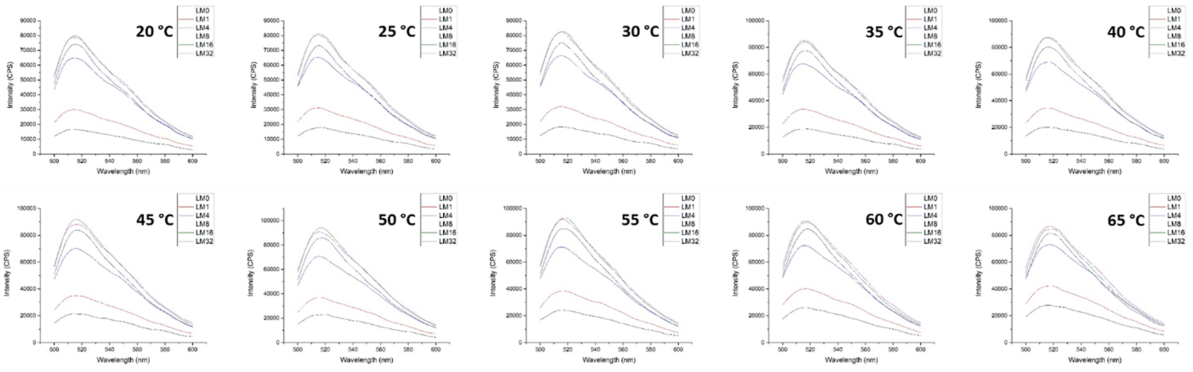

Figure S7. Fluorescence spectra of ligand concentration effect on sisomicin binding towards annealed RNA repeats at 20 – 65 °C. Annealed FM1 r(CUG)$_{10}$ RNA sample concentration 200 nM and volume size 150 µL; Sisomicin addition volume size 1.5 µL with ligand to RNA molar ratios of 0, 1, 4, 8, and 16; Fluorescence setting: excitation at 485 nm, emission at 500 – 600 nm, and slit width of 3 nm.

Part IV. Fluorescence characterization on denatured CUG RNA repeats

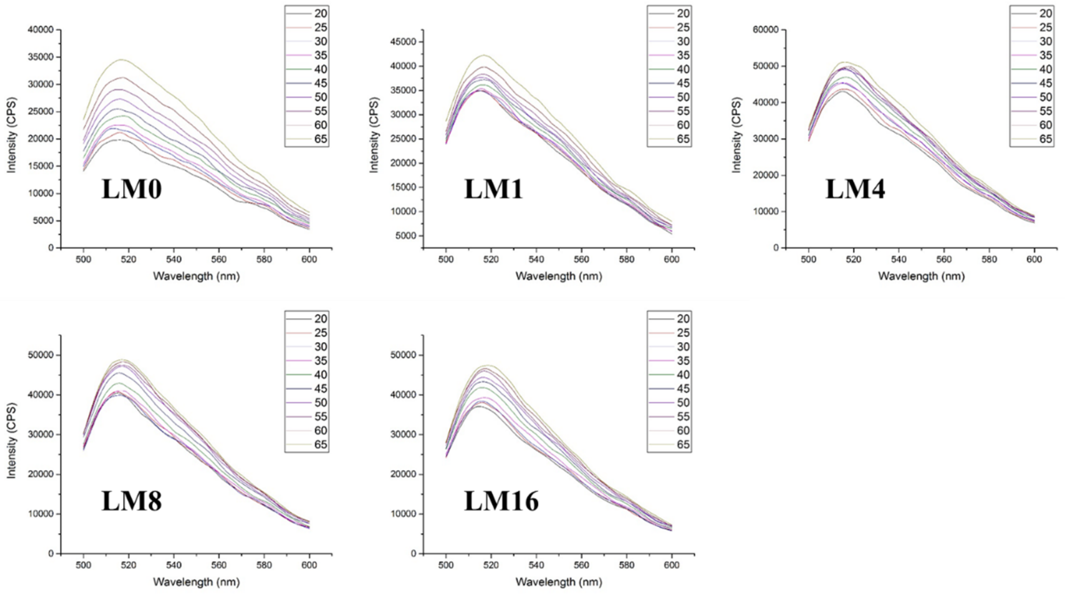

Figure S8. Fluorescence spectra of temperature effect on sisomicin binding towards denatured RNA repeats with different L/M. Denatured FM1 r(CUG)$_{10}$ RNA sample concentration 200 nM and volume size 150 µL; Sisomicin addition volume size 1.5 µL with ligand to RNA molar ratios – L/M at 0, 1, 4, 8, and 16; Fluorescence setting: excitation at 485 nm, emission at 500 – 600 nm, and slit width of 3 nm at 20 – 65 °C.

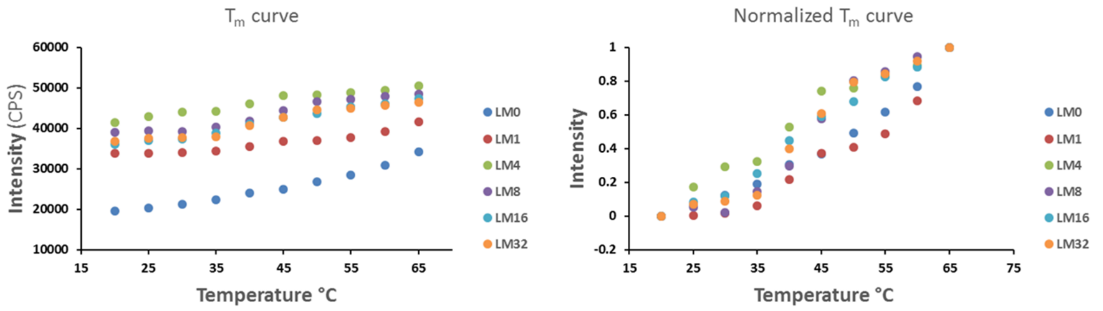

Figure S9. T_m curves for sisomicin binding towards denatured RNA with different L/M. Denatured FM1 r(CUG)$_{10}$ RNA sample concentration 200 nM and volume size 150 µL; Sisomicin addition volume size 1.5 µL with ligand to RNA molar ratios – L/M at 0, 1, 4, 8, and 16; Fluorescence setting: excitation at 485 nm, emission at 500 – 600 nm, and slit width of 3 nm at 20 – 65 °C.

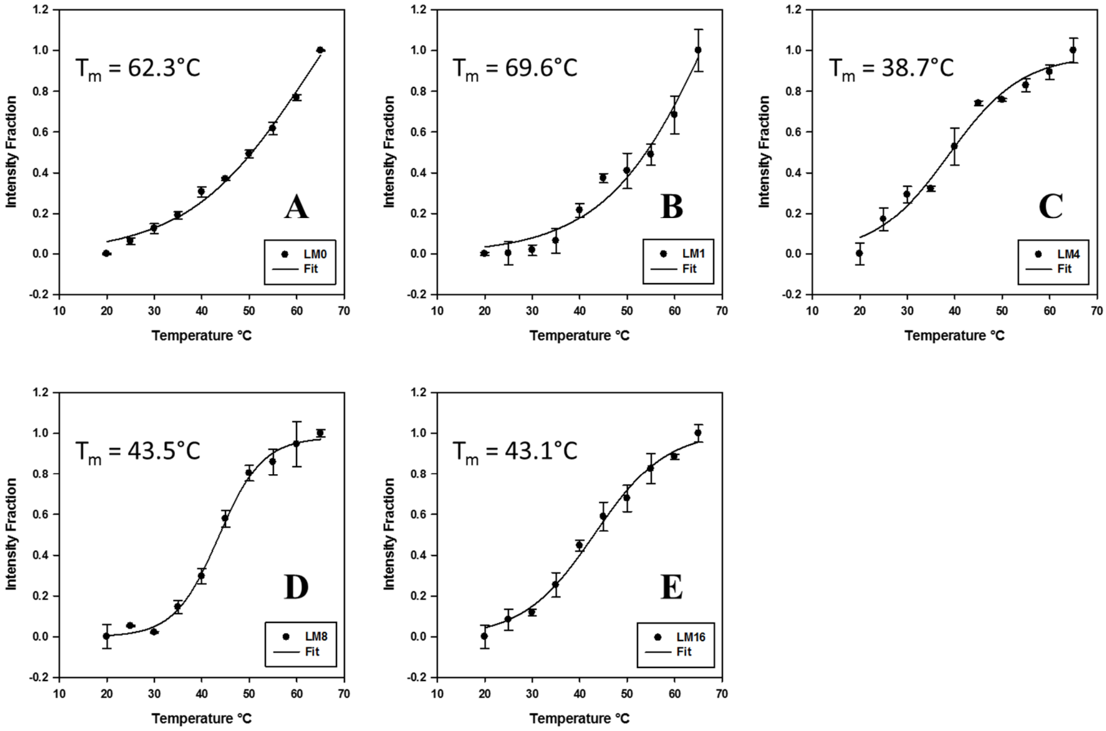

Figure S10. Sigmoidal fittings of T_m curves for titrating sisomicin against denatured FM1 r(CUG)$_{10}$: (A – E) T_m at ligand to RNA molar ratios of 0, 1, 4, 8, and 16. Denatured FM1 r(CUG)$_{10}$ RNA sample concentration 200 nM and volume size 150 µL; Ligand addition volume size 1.5 µL; Fluorescence setting: excitation at 485 nm, emission at 500 – 600 nm, and slit width of 3 nm in a range of 20 – 65 °C.

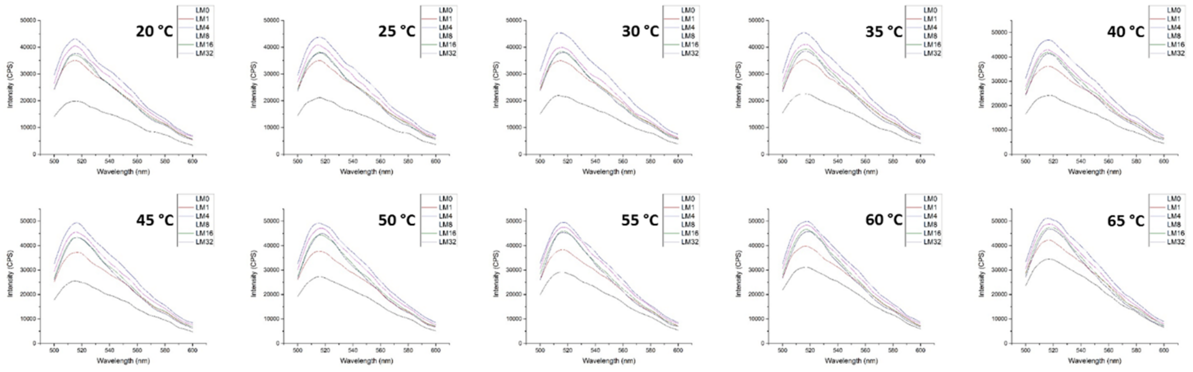

Figure S11. Fluorescence spectra of ligand concentration effect on sisomicin binding towards denatured RNA repeats at 20 – 65 °C. Denatured FM1 r(CUG)$_{10}$ RNA sample concentration 200 nM and volume size 150 µL; Sisomicin addition volume size 1.5 µL with ligand to RNA molar ratios of 0, 1, 4, 8, and 16; Fluorescence setting: excitation at 485 nm, emission at 500 – 600 nm, and slit width of 3 nm.

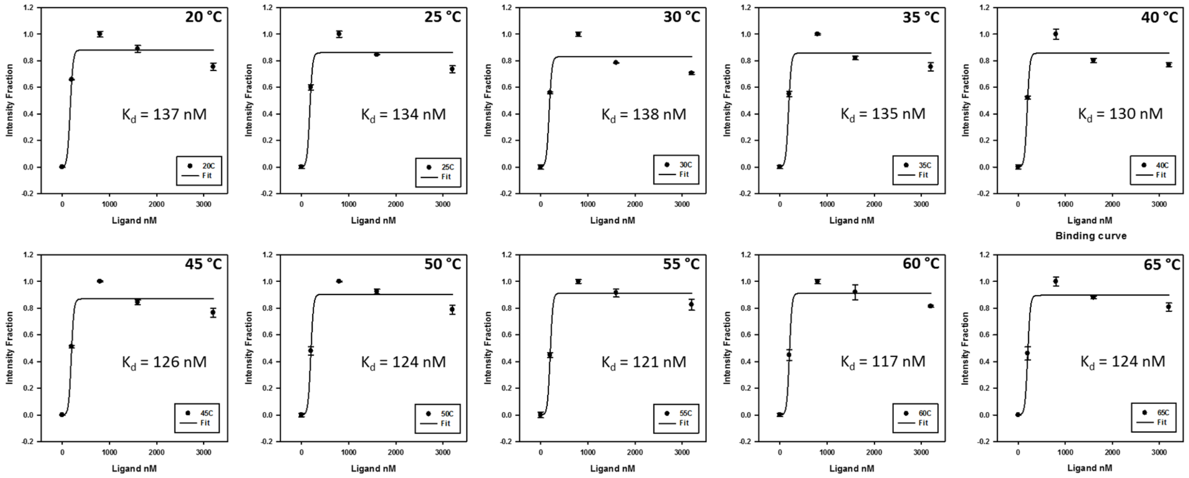

Figure S12. Sigmoidal data fitting of sisomicin binding towards denatured RNA repeats and calculated K_d values at 20 – 65 °C. Denatured FM1 r(CUG)$_{10}$ RNA sample concentration 200 nM and volume size 150 µL; Sisomicin addition volume size 1.5 µL with ligand to RNA molar ratios of 0, 1, 4, 8, and 16; Fluorescence setting: excitation at 485 nm, emission at 500 – 600 nm, and slit width of 3 nm.
